# Wastewater treatment evaluation for enterprises based on fuzzy-AHP comprehensive evaluation: a case study in industrial park in Taihu Basin, China

**DOI:** 10.1186/s40064-016-2523-8

**Published:** 2016-06-27

**Authors:** Wei Hu, Guangbing Liu, Yong Tu

**Affiliations:** 1 Jiangsu Key Laboratory of Environmental Engineering, Jiangsu Provincial Academy of Environmental Science, Nanjing University of Science & Technology, Nanjing, 210036 China; 2 Jiangsu Academy of Environmental Industry and Technology, Nanjing University of Science & Technology, Nanjing, 210036 China

**Keywords:** Analytic hierarchy process (AHP), Fuzzy comprehensive evaluation (FCE), Wastewater treatment evaluation, Evaluating index, Membership function, Taihu basin

## Abstract

This paper applied the fuzzy comprehensive evaluation (FCE) technique and analytic hierarchy process (AHP) procedure to evaluate the wastewater treatment for enterprises. Based on the characteristics of wastewater treatment for enterprises in Taihu basin, an evaluating index system was established for enterprise and analysis hierarchy process method was applied to determine index weight. Then the AHP and FCE methods were combined to validate the wastewater treatment level of 3 representative enterprises. The results show that the evaluation grade of enterprise 1, enterprise 2 and enterprise 3 was *middle, good* and *excellent,* respectively. Finally, the scores of 3 enterprises were calculated according to the hundred-mark system, and enterprise 3 has the highest wastewater treatment level, followed by enterprise 2 and enterprises 1. The application of this work can make the evaluation results more scientific and accurate. It is expected that this work may serve as an assistance tool for managers of enterprise in improving the wastewater treatment level.

## Background

As the third largest freshwater lake in China, Taihu plays an important role in flood control, water supply, and fishery in Yangtze River Delta region. Due to the rapid development of economy in Taihu Lake Basin and different construction level of surrounding industrial park, a large number of wastewater was discharged into the Taihu lakes, which has serious harmful on the water quality of Taihu. Therefore, it is important to carry out the wastewater treatment evaluation of enterprise in industrial park, which has practical significance for enterprise to strengthen pollution control.

In order to accurately evaluate the level of wastewater treatment of enterprise, it is important to choose the scientific and effective methods. Fuzzy Theory is a method used to study and deal with fuzzy phenomena; it has lasted 50 years since it was first proposed by Zadeh (1965, 1975). With development of fuzzy comprehensive evaluation method, the Fuzzy Analytical Hierarchy Process (FAHP) was developed based on the theory of FCE and AHP, and has been extensively applied in the fields of safety and risk assessment (Lai et al. 2015; Li et al. 2015; Chen et al. 2014; Padma and Balasubramanie 2011), technological comparison (Chen et al. 2015; Liu et al. 2014; Gim and Kim 2014), environmental evaluation (Shi et al. 2014; Feng et al. 2014), market decisions (Lee et al. 2011; Ho 2012; Li et al. 2014; Tsai et al. 2008), appearance products design (Hsiao and Ko 2013; Hsiao 1995, 1998; Hsiao and Chen 1997; Hsiao and Wang 1998), and facility location applications (Choudhary and Shankar 2012; Kaya and Kahraman 2010; Kabir and Sumi 2014) etc. However, it is rarely applied in the field of wastewater treatment evaluation in industrial park.

On the basis of this background, this paper adopted fuzzy-AHP comprehensive evaluation approach to study the wastewater treatment evaluation for enterprises in Taihu Basin, China. It is expected that this work may serve as an assistance tool for managers of enterprise in improving the wastewater treatment level.

## Theoretical background

### Fuzzy comprehensive evaluation

Fuzzy comprehensive evaluation steps included five parts: establishing the evaluation parameter, determining factor weight, constructing a parameter evaluation, building a single factor evaluation matrix and conducting fuzzy evaluation, as follows:

1. Establishing the evaluation parameter
  For fuzzy evaluation, factors affected the evaluation parameter should first be constructed. If the affected factors are u_1_, u_2_, … u_m_, the parameters set can define: ${\text{U}} = \left\{ {u_{1} ,u_{2} , \ldots ,u_{m} } \right\} = \left\{ {u_{i} }\right\}, \quad ({\text{i}} = 1,2, \ldots ,{\text{m}}).$
2. Determining factor weight
  Each factor has a different impact on the parameters. So the factors have different weights for parameter values. The set composed of various weights of all factors is called the factor weight set, which is represented as A = {*a*_1_, *a*_2_, …, *a*_*n*_}. The weight of each factor must satisfy Eq. (1).
  1 $$\mathop \sum \limits_{i = 1}^{n} a_{i} = 1,\,a_{i} \ge 0,\quad {\text{i}} = 1,2,\, \ldots ,{\text{n}}$$
  There are many methods to confirm the index weight, such as the expert evaluation method, least squares estimation, AHP method and etc. The AHP is much more widely used by the analyzers. This method can analyze the important degree of the index more logically than other methods, and correspondingly the result disposed by mathematics are more reliable. In this study, AHP method was used to determining the factor weight.
3. Constructing a parameter evaluation
  An evaluation set is the set of various possible evaluation results given by evaluators for the evaluation objects, shown as ${\text{V}} = \left\{ {v_{1} ,v_{2} , \ldots ,v_{n} } \right\} = \left\{ {v_{j} } \right\},({\text{j}} = 1,2, \ldots ,{\text{n}})$, where $v_{n}$ is the grade of evaluation. The purpose of fuzzy evaluation is to obtain an optimal evaluation result from the evaluation set.
4. Building a single factor evaluation matrix
  A single factor fuzzy evaluation system was used to determine the membership of an evaluation object. The evaluation result of No. i factor U_i_ can be expressed as:
  2 $$\begin{array}{*{20}c} {{\text{R}}_{1} = \left( {r_{11} ,r_{12} , \ldots ,r_{1n} } \right)} \\ {{\text{R}}_{2} = (r_{21} ,r_{22} , \ldots ,r_{2n} ) } \\ \vdots \\ {{\text{R}}_{m} = (r_{m1} ,r_{m2} , \ldots ,r_{mn} )} \\ \end{array}$$
  where *r*_*mn*_ represents the membership degree of j factors to comment V_i_, R_m_ is called single factor evaluation set.
5. Conducting fuzzy evaluation
  If the fuzzy evaluation matrix of an evaluation object is:
  3 $$R = \left[ {\begin{array}{*{20}c} {R_{1} } \\ {R_{2} } \\ \cdots \\ {R_{m} } \\ \end{array} } \right] = \left[ {\begin{array}{*{20}c} {r_{11} } & {r_{12} } & \cdots & {r_{1n} } \\ {r_{21} } & {r_{22} } & \cdots & {r_{2n} } \\ \cdots & \cdots & \cdots & \cdots \\ {r_{m1} } & {r_{m2} } & \cdots & {r_{mn} } \\ \end{array} } \right]$$
  Then the comprehensive fuzzy evaluation matrix is:
  4 $${\text{B}} = {\text{A}}\cdot{\text{R}} = \left( {a_{1} ,a_{2} , \cdots ,a_{n} } \right) \cdot \left[ {\begin{array}{*{20}c} {r_{11} } & {r_{12} } & \cdots & {r_{1n} } \\ {r_{21} } & {r_{22} } & \cdots & {r_{2n} } \\ \cdots & \cdots & \cdots & \cdots \\ {r_{m1} } & {r_{m2} } & \cdots & {r_{mn} } \\ \end{array} } \right] = (b_{1} ,b_{2} , \ldots ,b_{n} )$$
  where B is the evaluation result based on all factors in the index system U. In the above equation, the symbol “·” represent fuzzy composition. This study will use M(·, +) algorithm to work out various evaluation results for comparison and analysis.

### Analytic hierarchy process

Analytical Hierarchy Process (AHP), first introduced by Saaty (1980), is a systematic approach to solving complex and multi-level decision-making problems. Based on the expert judgments, the criteria are compared in a pairwise fashion to assess how they contribute to the target. However, in many cases the preference model of the human decision-maker is uncertain and fuzzy, and the comparison ratios are relatively difficult to be provided. The decision-maker may be uncertain due to incomplete information or knowledge, inherent complexity and uncertainty within the decision environment. Therefore, some researchers have improved the fuzzy pairwise comparison judgements. In Rezaei’s study (Rezaei et al. 2013), they improve a fuzzy AHP and then apply it using the pairwise comparisons of three experts to evaluate the entrepreneurship orientation of 59 small to medium-sized enterprises (SMEs) and rank the firms based on their entrepreneurship orientation score. In Mikhailov’s study (Mikhailov 2003), a new approach for deriving priorities from fuzzy pairwise comparison judgements is proposed, based on α-cuts decomposition of the fuzzy judgements into a series of interval comparisons. Meanwhile, a modification of the linear fuzzy preference programming method is also proposed to derive priorities directly from fuzzy judgements, without applying α-cut transformations. Both proposed methods are illustrated by numerical examples and compared to some of the existing fuzzy prioritisation methods. Leung and Cao (2000) proposes a fuzzy consistency definition with consideration of a tolerance deviation, and determined the fuzzy local and global weights via the extension principle.

The AHP method can be divided into the five steps:

Step 1: Defining the decision-making problem.

Step 2: Constructing a hierarchical structure.

Step 3: Building a pairwise comparison matrix.

Step 4: Calculate eigenvalues.

Step 5: Conformance test.

A consistency ratio (CR) must be computed [Eq. (5)] to check the discordances between the pairwise comparisons and the reliability of the obtained weights. The value must be <0.1 to be accepted; otherwise, it is necessary to recalculate the weight.

5 $${\text{CR}} = \frac{{C{\text{I}}}}{{R{\text{I}}}}$$

where RI is a random index represented the consistency of a randomly generated pairwise comparison matrix. Its reference standard, shown in Table 1, was computed and recommended by Saaty (1980). CI represents the consistency index computation:

**Table 1:** Table of random indexes

n	1	2	3	4	5	6	7	8	9	10	11	12	13	14	15
RI	0	0	0.58	0.90	1.12	1.24	1.32	1.41	1.45	1.49	1.51	1.48	1.56	1.57	1.58

6 $${\text{CI}} = \frac{{\lambda_{max} - {\text{n}}}}{n - 1}$$

where *λ*_*max*_ is the largest eigenvalue of the matrix, n is matrix order (number of parameters).

## Case study

### Construction of evaluation index system

The wastewater treatment for enterprises evaluation system is a big system, which can be divided into economy, society and environment subsystems. Due to the abundant factors contained, it is necessary to choose several representative factors as evaluation index. The choice of index should pay attention to the comprehensive, representative, reasonable and realistic aspects of factor. Both comprehensive and particular features of the wastewater treatment for enterprises should be indicated.

According to the above principles, and combined with the characteristics of industrial wastewater treatment in Taihu Basin, 12 index of wastewater treatment evaluation system for enterprises was constructed from three aspects (environmental protection benefit, resource utilization benefit and recycling benefit) in this paper, as shown in Table 2.

**Table 2:** The wastewater treatment evaluation index system for enterprises

The first level	The second level (criteria)	The third level (alternatives)
T: Wastewater treatment evaluation for enterprises in industrial park	U_1_: Environmental protection benefit	u_11_: COD effluent concentration
		u_12_: NH_3_-N effluent concentration
		u_13_: TP effluent concentration
		u_14_: TN effluent concentration
		u_15_: Effluent colority
	U_2_: Resource utilization benefit	u_21_: Unit product water consumption
		u_22_: Unit product wastewater discharge
		u_23_: Wastewater treatment cost per ton
		u_24_: Operating load of sewage treatment
	U_3_: Recycling benefit	u_31_: Recycling rate of industrial water
		u_32_: Reuse rate of tail water
		u_33_: Stability compliance rate of wastewater treatment

### Data collection and analysis

Measurement methods of COD, NH_3_-N, TP, TN and colority pollutant concentration are carried out in accordance with the Chinese national standard method, which are shown in Table 3.

**Table 3:** Table of determination method

Serial number	Index	Measurement method/calculation method	Detection limits/unit	Chinese national standard
1	COD (chemical oxygen demand)	Dicolorityte method	10 mg/L	GB11914-89
2	Ammonium nitrogen (NH_3_-N)	Salicylic acid spectrophotometry	0.01 mg/L	GB7481-87
3	Total phosphorus (TP)	Ammonium molybdate spectrophotometric method	0.01 mg/L	GB11893-89
4	Total nitrogen (TN)	Alkaline potassium persulfate digestion UV spectrophotometric method	0.05 mg/L	GB11894-89
5	Colority	Dilution multiple method	Dimensionless	GB11903-89
6	Unit product water consumption	Water consumption/output of qualified products	m^3^/t	Empirical calculation method
7	Unit product wastewater discharge	Wastewater discharge/output of qualified products	m^3^/t	Empirical calculation method
8	Wastewater treatment cost per ton	Wastewater treatment cost/Wastewater discharge	RMB/t	Empirical calculation method
9	Operating load of sewage treatment	Actual wastewater treatment quantity/designed wastewater treatment quantity	m^3^/m^3^	Empirical calculation method
10	Recycling rate of industrial water	Repeated utilization of water quantity/(fresh water supplement + repeated utilization of water quantity)	%	Empirical calculation method
11	Reuse rate of tail water	Reuse quantity of tail water/water consumption	%	Empirical calculation method
12	Stability compliance rate of wastewater treatment	Stability compliance number/total monitoring number	%	Empirical calculation method

Effluent pollutant concentration of COD, NH_3_-N, TP, TN and colority comes from the monthly routine monitoring data of enterprises, while unit product water consumption, unit product wastewater discharge, wastewater treatment cost per ton, operating load of sewage treatment, recycling rate of industrial water and reuse rate of tail water comes from the statistical data of enterprise. The statistical results of 12 indexes of 3 enterprises were seen in Tables 4, 5 and 6 in 2014. It can be seen from the Tables 4, 5 and 6 that the average value of the 12 indexes of enterprise 3 was relatively low, followed by enterprise 2 and enterprise 1.

**Table 4:** Summary of index actual values for enterprise 1 in 2014

Index	Jan	Feb	Mar	Apr	May	Jun	Jul	Aug	Sep	Oct	Nov	Dec	Average
COD effluent concentration	347	358	388	410	401	329	337	420	360	372	380	313	368
NH_3_-N effluent concentration	29.4	27.8	36.6	26.3	28.7	25.8	28.4	33.5	29	32.5	34.7	37.1	30.8
TP effluent concentration	1.7	1.8	2.3	2	1.6	2.7	2.8	1.8	2.5	2.1	2.3	1.5	2.1
TN effluent concentration	50.7	41.1	37.2	34.6	35.6	53.3	50.5	42.4	48	47	38.5	45.4	43.7
Effluent colority	80	50	60	50	60	50	60	60	70	50	70	60	60
Unit product water consumption	213.7	279.3	203.5	219.7	243.2	199.3	195.5	286.5	217.8	225.4	268.6	190.8	228.6
Unit product wastewater discharge	161.1	150.6	140.8	205.6	198.5	133.7	173.6	224.2	139.5	193.4	152.4	183.3	171.4
Wastewater treatment cost per ton	1.12	0.8	0.91	1.04	1.59	1.16	1.25	1.06	1.21	1.37	1.17	1.23	1.16
Operating load of sewage treatment	72	54	57	63	46	81	54	72	59	48	65	48	60
Recycling rate of industrial water	20	32	27	23	36	16	31	23	24	30	22	17	25
Reuse rate of tail water	63	56	89	82	64	73	58	61	78	82	69	64	70
Stability compliance rate of wastewater treatment	98.8	99	98.4	99.2	99.4	98.6	98.9	98.9	99.3	98.8	98.5	99.1	98.9

**Table 5:** Summary of index actual values for enterprise 2 in 2014

Index	Jan	Feb	Mar	Apr	May	Jun	Jul	Aug	Sep	Oct	Nov	Dec	Average
COD effluent concentration	248	216	194	173	224	256	237	208	183	165	151	195	204
NH_3_-N effluent concentration	18.5	19.2	23.6	22.6	24.8	26.3	16.4	17.9	18.8	19.5	27.2	15.9	20.9
TP effluent concentration	1.2	1.5	0.7	0.8	1.1	0.9	0.6	1.8	2	1.4	0.8	1.7	1.2
TN effluent concentration	26.7	23.7	35.1	39	41.5	25.4	22.9	29.3	33.5	38.7	36.2	30.6	31.9
Effluent colority	30	40	40	50	60	40	30	30	40	40	40	50	40
Unit product water consumption	125.3	148.7	203.6	224.8	131	154.7	160.4	138.5	134.6	221.5	178.3	142	163.6
Unit product wastewater discharge	117.5	158.4	138.9	126.5	190.3	105.8	148.3	170.6	163.5	98.7	102.5	115.6	136.4
Wastewater treatment cost per ton	1.38	1.49	1.63	1.32	1.17	1.06	1.43	1.85	1.42	1.51	1.47	1.3	1.42
Operating load of sewage treatment	58	70	62	44	38	49	45	36	38	62	53	44	50
Recycling rate of industrial water	37	36	26	22	32	30	28	25	21	36	40	25	30
Reuse rate of tail water	0	0	0	0	0	0	0	0	0	0	0	0	0
Stability compliance rate of wastewater treatment	99.5	99.2	99.8	99.4	98.8	99.3	99.7	98.9	99.1	99.5	99.3	99.2	99.3

**Table 6:** Summary of index actual values for enterprise 3 in 2014

Index	Jan	Feb	Mar	Apr	May	Jun	Jul	Aug	Sep	Oct	Nov	Dec	Average
COD effluent concentration	89	81	172	111	153	110	68	99	140	124	132	163	120
NH_3_-N effluent concentration	9.5	16.4	7.5	11.1	8.1	18.8	10.4	14.3	17.9	10.8	15.2	10.1	12.5
TP effluent concentration	0.8	0.4	0.3	0.5	1.2	1.4	1	0.3	0.4	1.2	1.6	0.7	0.8
TN effluent concentration	27	11.3	24.5	18.9	17.6	12.1	21.9	13.7	27.2	29.8	23.5	15	20.2
Effluent colority	30	10	20	40	20	10	20	30	20	20	20	10	20
Unit product water consumption	190	117.4	121	124.9	164.6	207.9	211.3	141.1	128.5	135.2	146.8	111.7	150
Unit product wastewater discharge	69.4	134.2	79.2	111.9	62.3	127.4	102.5	66.1	154	90.1	81.7	122	100
Wastewater treatment cost per ton	1.42	1.54	1.29	1.18	1.59	1.97	1.63	1.75	1.44	1.56	1.53	1.61	1.54
Operating load of sewage treatment	19	13	24	20	28	13	36	37	33	19	14	45	25
Recycling rate of industrial water	20	18	15	26	15	26	31	16	22	12	11	27	20
Reuse rate of tail water	0	0	0	0	0	0	0	0	0	0	0	0	0
Stability compliance rate of wastewater treatment	99.8	99.6	100	99.9	99.7	99.8	99.5	100	99.9	99.6	99.7	100	99.8

### Grading standard

According to the comprehensive consideration of the actual situation of enterprise wastewater treatment in the industrial park of the Taihu basin, the evaluating set is divided into four grades in this study: *excellent, good, middle, bad*. The grading standard is based on the accessing standard of sewage treatment plant, the field survey, the expert consultation and the cleaner production evaluation index system of the industry in China.

Usually, for the normal operation of the sewage treatment plant, the enterprise’s wastewater must be pretreatment before entering in the sewage treatment plant. Thus the influent concentration of pollutants has an accessing standard, and the accessing standard of COD, NH_3_-N, TP, TN and colority of sewage treatment plant were 500, 45, 8, 70 and 70 respectively in this study. Taking the COD as an example, the COD accessing concentration of sewage treatment plant must be less than 500 mg/L, or the sewage treatment plant will be overloaded operation if exceed 500 mg/L. According to the many year operation experiences of sewage treatment plant, the lower influent concentration of COD were, the better treatment effect of sewage treatment plant achieved.

In addition, the expert consultation method was used to determine the grading standard. The designed table for expert consultation was shown in Table 7. 30 copies of the expert consultation form were sent and all of it was recovered. The statistical results of the 30 expert consultation was shown that 4 grading was chosen by 23 experts, 3 grading by 4 experts and 5 grading by 3 experts for question 1. Furthermore, in the 23 consultation table with choice of 4 grading, 20 experts believed that the grading standard of COD were appropriate for 100, 200, 300 and 400. So the COD grading standard was divided into four grades in this study*: excellent, good, middle, bad*, and the grading standard were 100, 200, 300 and 400 respectively.

**Table 7:** Designed table for expert consultation

Expert name	Work unit	Title
Question 1	What grading number do you think is appropriate? 3, 4, 5, or others? Please write down in the right blank place	
Question 2	According to the COD accessing standard of sewage treatment plant and the grading number determined in question 1, what values of each grading do you think is appropriate? Please write down the values of each grading in the right blank place	

Similarly, the grading standard of NH_3_-N, TP, TN and colority index can be obtained. At the same time, grading standard of unit product water consumption, unit product wastewater discharge, wastewater treatment cost per ton, operating load of sewage treatment, recycling rate of industrial water and reuse rate of tail water index were obtained by consulting Chinese printing and dyeing industry cleaner production evaluation index system and expert consultation results. Finally, the critical values of the grading standard in this study are shown in Table 8.

**Table 8:** Grading standard of wastewater treatment evaluation for enterprises

Index	Grading standard			
	Excellent	Good	Middle	Bad
COD effluent concentration	100	200	300	400
NH_3_-N effluent concentration	5	15	25	35
TP effluent concentration	1	2	3	4
TN effluent concentration	25	35	45	55
Effluent colority	10	30	50	70
Unit product water consumption	100	150	200	250
Unit product wastewater discharge	80	120	160	200
Wastewater treatment cost per ton	1	1.2	1.4	1.6
Operating load of sewage treatment	80	70	60	50
Recycling rate of industrial water	40	30	20	10
Reuse rate of tail water	40	30	20	10
Stability compliance rate of wastewater treatment	100	99.5	99	98.5

### Membership function

Since indices vary in range and dimension values, a unified standard is needed in the same evaluation system, which can be solved by membership function. In general, the membership degree of each level can be determined by the piecewise linear function in fuzzy mathematics and descending semi-trapezoid function was used in this study. According to the critical value of the grading standard (Table 3), the membership degrees of the twelve single evaluation factors to the grading level set were calculated applying the above calculating method given in “Fuzzy comprehensive evaluation” section, and the single-factor evaluation matrices were produced. Taking the enterprise 1 as an example, the membership degree of COD effluent concentration index is calculated as follows:

$$r_{1} = 0$$

$$r_{2} = 0$$

$$r_{3} = \frac{400 - 368}{400 - 300} = \frac{32}{100} = 0.32$$

$$r_{4} = \frac{368 - 300}{400 - 300} = \frac{68}{100} = 0.68$$

Therefore, the membership degree of the COD effluent concentration was (0, 0, 0.32, 0.68). Similarly, the membership degree of other’s index can be obtained. The evaluation matrices of indexes were then formed in follows:

$${\text{R}}_{1} = \left[ {\begin{array}{*{20}c} 0 & 0 & {0.32} & {0.68} \\ 0 & 0 & {0.42} & {0.58} \\ 0 & {0.9} & {0.1} & 0 \\ 0 & {0.13} & {0.87} & 0 \\ 0 & 0 & {0.5} & {0.5} \\ \end{array} } \right]$$

$${\text{R}}_{2} = \left[ {\begin{array}{*{20}c} 0 & 0 & {0.572} & {0.428} \\ 0 & 0 & {0.715} & {0.285} \\ {0.2} & {0.8} & 0 & 0 \\ 0 & 0 & 1 & 0 \\ \end{array} } \right]$$

$${\text{R}}_{3} = \left[ {\begin{array}{*{20}c} 0 & {0.5} & {0.5} & 0 \\ 1 & 0 & 0 & 0 \\ 0 & 0 & {0.8} & {0.2} \\ \end{array} } \right]$$

### Weight analysis

30 peoples including college students from wastewater treatment-related majors, scholars and experts filled in the questionnaire. The determination of weight is built into a pairwise comparison matrix by AHP. The total sum of what the coefficients related to the pairwise comparison matrix multiply each part’s weight is the λ value of each part, and it is incorporated to calculate λ_max_. Taking T-U judgment matrix as an example, the calculation process is shown in Table 9.

**Table 9:** Weight of T-U judgment matrix using square root method

T	U_1_	U_2_	U_3_	M = ∏*M*_*ij*_	$W_{I}^{\prime } = \sqrt[n]{M}$	*W*_*i*_ = *W*_*i*_^′^/∑*W*_*i*_^′^	(AW)_i_	(AW)_i_/W_i_
U_1_	1	1	2	2	1.260	0.413	1.260	3.051
U_2_	1	1	1	1	1	0.327	1	3.058
U_3_	1/2	1	1	0.5	0.794	0.260	0.794	3.054
–	–	–	–	Total Σ	3.054	1.000	–	9.163

By using of square root method, the maximum eigenvalue (λ_max_) is obtained:

$$\lambda_{max} = \mathop \sum \limits_{i = 1}^{n} \frac{{b_{ij} W}}{{nW_{i} }} = \frac{1}{3} \times 9.163 = 3.054.$$

Consistency index is:

$$CI = \frac{{\lambda_{max} - {\text{n}}}}{n - 1} = \frac{3.054 - 3}{3 - 1} = 0.027.$$

Random consistency rate is:

$$CR = \frac{CI}{RI} = \frac{0.027}{0.58} = 0.047 < 0.1.$$

Similarly, each index weight can be determined, random consistency rate can also be confirmed. The calculating process is omitted, and results are shown in Table 10. Due to all the random consistency rates are less than 0.1, so all the judgment matrix are satisfactory. Therefore, the index weight vectors are: A = (0.413 0.327 0.260); A_1_ = (0.168 0.168 0.306 0.306 0.052), A_2_ = (0.227 0.227 0.423 0.123), A_3_ = (0.25 0.25 0.5), respectively.

**Table 10:** Comparison matrix and the consistency test

Index	Comparison matrix B	Weight A_i_	Consistency test
u_11_	1 1 1/2 1/2 4	0.168	*λ*_*max*_ = 5.0354
u_12_	1 1 1/2 1/2 4	0.168	*CI* = 0.00885
u_13_	2 2 1 1 5	0.306	*RI* = 1.12
u_14_	2 2 1 1 5	0.306	*CR* = *CI*/*RI* = 0.0079 < 0.1, meets the requirements of consistency
u_15_	1/4 1/4 1/5 1/5 1	0.052	
u_21_	1 1 1/2 2	0.227	*λ*_*max*_ = 4.01
u_22_	1 1 1/2 2	0.227	*CI* = 0.003333
u_23_	2 2 1 3	0.423	*RI* = 0.90
u_24_	1/2 1/2 1/3 1	0.123	*CR* = *CI*/*RI* = 0.0037 < 0.1, meets the requirements of consistency
			*λ*_*max*_ = 3
u_31_	1 1 1/2	0.25	*CI* = 0
u_32_	1 1 1/2	0.25	*RI* = 0.58
u_33_	2 2 1	0.5	*CR* = *CI*/*RI* = 0 < 0.1, meets the requirements of consistency

### Fuzzy comprehensive evaluation

1. First order fuzzy comprehensive evaluation.
  Taking enterprise 1 as an example, first order fuzzy comprehensive evaluation on B_1_ (environmental protection benefit) factor can be calculated as follow: $$B_{1} = A_{1} R_{1} = \left( {0. 1 6 8 { }0. 1 6 8 { }0. 30 6 { }0. 30 6 { }0.0 5 2} \right)\left[ {\begin{array}{*{20}c} 0 & 0 & {0.32} & {0.68} \\ 0 & 0 & {0.42} & {0.58} \\ 0 & {0.9} & {0.1} & 0 \\ 0 & {0.13} & {0.87} & 0 \\ 0 & 0 & {0.5} & {0.5} \\ \end{array} } \right]\, = \left( {0 \, 0. 3 1 5 { }0. 4 4 7 { }0. 2 3 8} \right)$$
  Similarly, we got the evaluation result of B_2_ (resource utilization benefit) and B_3_ (recycling benefit) through calculations:
  $$\eqalign{ & {B_2} = {A_2} \cdot {R_2} = (0.085\,\,0.338\,\,0.415\,\,0.162). \cr & {B_3} = {A_3} \cdot {R_3} = (0.25\,\,0.125\,\,0.525\,\,0.1). \cr}$$
2. Second order fuzzy comprehensive evaluation.
  The comprehensive evaluation of wastewater treatment for enterprises is calculated as: $${\text{B}} = {\text{A}}\cdot{\text{R}} = {\text{A}}\cdot\left[ {\begin{array}{*{20}c} {B_{1} } \\ {B_{2} } \\ {B_{3} } \\ \end{array} } \right] = (0. 4 1 3\text{ }0. 3 2 7\text{ }0. 2 60)\cdot\left[ {\begin{array}{*{20}c} 0 & {0.315} & {0.447} & {0.238} \\ {0.085} & {0.338} & {0.415} & {0.162} \\ {0.25} & {0.125} & {0.525} & {0.1} \\ \end{array} } \right]\, = \left( {0.0 9 3 { }0. 2 7 3 { }0. 4 5 7 { }0. 1 7 7} \right)$$.
  Through the above calculation, the evaluation grade of evaluation object is determined on maximum membership degree principle. The result shows that the probability of “*excellent*”, “*good*”, “*middle*” and “*bad*” is 0.093, 0.273, 0.457 and 0.177 respectively. According to the maximum membership degree principle, the evaluation result of the enterprise 1 is “*middle*.” Same to the calculating process of enterprise 1, the result vectors of other enterprise can be obtained, as shown in Table 11. It can be seen that evaluation grade of enterprise 1, enterprise 2 and enterprise 3 is *middle, good* and *excellent* respectively.

**Table 11:** Fuzzy comprehensive evaluation results of wastewater treatment evaluation for enterprises

Index	Membership degree				Evaluation grade
	Excellent	Good	Middle	Bad	
Enterprise 1	0.093	0.273	0.457	0.177	*Middle*
Enterprise 2	0.188	0.411	0.282	0.119	*Good*
Enterprise 3	0.451	0.240	0.106	0.202	*Excellent*
  And then calculate the value of comprehensive evaluation and determine the level of the evaluation, first, give the score of the set of evaluation according to the hundred-mark system, thus we can get the data of the set of evaluation by assign values: K = {95, 85, 75, 65}, finally, got the scores of comprehensive evaluation of enterprise 1, enterprise 2 and enterprise 3 as follows:
  $$V_{1} = {\text{B}} \times V^{T} = \left[ {0.093 0.273 0.457 0.177} \right] \times [95 85 75 65]^{T} = 77.8 .$$
  Similarly, V_2_ = 81.7, V_3_ = 84.3, V_3_ > V_2_ > V_1_, so we can think that the wastewater treatment evaluation result is enterprise 3 > enterprise 2 > enterprise 1.
  The validation of the procedure steps with experimental data was shown in Table 12. It can be seen from data that enterprise 3 has the smallest values for unit product COD, NH_3_-N, TP and TN discharge compared to enterprise 1 and enterprise 2, which means that the fuzzy AHP evaluation results of the enterprise 3 was in a better level, followed by enterprise 2 and enterprise 1. Thus the fuzzy AHP procedure steps were fulfilled by the experimental data.

**Table 12:** Validation of the procedure steps with experimental data

Index	Enterprise 1	Enterprise 2	Enterprise 3
Unit product COD discharge (kg/t)	84.7	32.8	17.5
Unit product NH_3_-N discharge (kg/t)	7.0	3.5	1.9
Unit product TP discharge (kg/t)	0.5	0.2	0.1
Unit product TN discharge (kg/t)	9.9	5.3	3.0
3. Fuzzy AHP results compared with real situation.
  Unit product COD, NH_3_-N, TP, TN charge and unit product sewage charge were used to characterize the actual situation of wastewater treatment effect for enterprise in industrial Park in China. In general, the lower unit product COD (NH_3_-N, TP, TN) charge or unit product sewage charge, the better wastewater treatment effect of enterprise achieved. The unit product pollutant charge for enterprise was shown in Table 13. As can be seen from the Table 13, enterprise 3 has the smallest values for all indexes, with the largest values for enterprise 1. It means that the wastewater treatment effect of enterprise 3 is the best, followed by enterprise 2 and enterprise 1. On the other hand, the results indicates that the actual situation of wastewater treatment effect is corresponds to the experimental results.

**Table 13:** Unit product pollutant charge for enterprise

Index	Enterprise 1	Enterprise 2	Enterprise 3
Unit product COD charge (RMB/t)	98.3	77.9	54.4
Unit product NH_3_-N charge (RMB/t)	47.6	30.7	24.5
Unit product TP charge (RMB/t)	12.5	8.6	6.3
Unit product TN charge (RMB/t)	10.2	5.4	4.7
Unit product sewage charge (RMB/t)	185.6	143.8	110.5

## Conclusions and future research

An integrated framework using a fuzzy comprehensive evaluation method and an AHP procedure was proposed and applied to wastewater treatment evaluation for enterprise in Taihu Basin, China. The main results of this study are summarized in the following points.

1. According to the characteristics of industrial wastewater treatment in Taihu Basin, 12 index of wastewater treatment evaluation system for enterprises was constructed from three aspects (environmental protection benefit, resource utilization benefit and recycling benefit).
2. The index weight was calculated according to AHP theory. Calculation results reflected that weight vectors were: A = (0.413 0.327 0.260); A_1_ = (0.168 0.168 0.306 0.306 0.052), A_2_ = (0.227 0.227 0.423 0.123) and A_3_ = (0.25 0.25 0.5), respectively.
3. Fuzzy comprehensive evaluation results shown that the probability of “*middle*”, “*good*”, “*bad*” and “*excellent*” is 0.457 0.273, 0.177 and 0.093 respectively for enterprise 1. According to the maximum membership degree principle, the comprehensive evaluation result of the enterprise 1 is “*middle*.” Similarly, the evaluation grade of enterprise 2 and enterprise 3 is *good* and *excellent* respectively.

In future research, other MCDM and fuzzy approaches can be applied to assess the wastewater treatment evaluation for enterprises including ELECTRE (Benayoun et al. 1966), TOPSIS (Hawang and Yoon 1981), BWM (Birman and Wenzl 1989; Murakami 1987), VIKOR (Opricovic 1998) and so on. We think that the field of innovation and entrepreneurship can benefit from the experiences with fuzzy methods gained in the engineering sciences. Finally, we believe that fuzzy AHP approaches can be promoted in the wastewater treatment evaluation for enterprises in industrial park in China.
